# Supplementary material for: De novo lipogenesis fuels adipocyte autophagosome and lysosome membrane dynamics
Source: Nat Commun. 2023 Mar 13;14:1362. doi: 10.1038/s41467-023-37016-8 (PMC10011520; doi:10.1038/s41467-023-37016-8)
Supplement: Supplementary file 1 — Supplementary Information [file 41467_2023_37016_MOESM1_ESM.pdf]

Supplementary Table 1: Primer Sequences used for qPCR analyses

	Forward sequence (5'→3')	Reverse sequence (5'→3')
18s	CGA ACG TCT GCC CTA TCA ACT T	CCG GAA TCG AAC CCT GAT T
Acly	TGG TGG AAT GCT GGA CAA	GCC CTC ATA GAC ACC ATC TG
Acss2	GTG GAT GAA AGG AGC AAC TAC A	GCC CTC CCA GTA AAA AGC AAC T
Atg7	GTT CGC CCC CTT TAA TAG TGC	TGA ACT CCA ACG TCA AGC GG
Atg9a	CAG TTT GAC ACT GAA TAC CAG CG	AAT GTG GTG CCA AGG TGA TTT
B2m	TTC TGG TGC TTG TCT CAC TGA	CAG TAT GTT CGG CTT CCC ATT C
Becn1	ATG GAG GGG TCT AAG GCG TC	TCC TCT CCT GAG TTA GCC TCT
Fasn	GGAGGTGGTGATAGCCGGTAT	TGGGTAATCCATAGAGCCCAG
Glut4	CAT TCC CTG GTT CAT TGT GG	GAA GAC GTA AGG ACC CAT AGC
Map1lc3a	GAC CGC TGT AAG GAG GTG C	CTT GAC CAA CTC GCT CAT GTT A
Map1lc3b	TTA TAG AGC GAT ACA AGG GGG AG	CGC CGT CTG ATT ATC TTG ATG AG
Mitf	AAG TCG GGG AGG AGT TTC ACG	GGA GCT TAA CGG AGG CTT GGA
Plin1	ATG TCA ATG AAC AAG GGC CCA ACC	TGG TGC TGT TGT AGG TCT TCT GGA
Plin2	CAG CCA ACG TCC GAG ATT G	CAC ATC CTT CGC CCC AGT T
Pparg2	ATGGGTGAAACTCTGGGAG	GTGGTCTTCCATCACGGAGA
Sqstm1	GAC AGC CAG AGG AAC AGA TGG A	AGA GCT TGG CCC TTC CGA TT
Tfe3	TGC GTC AGC AGC TTA TGA GG	AGA CAC GCC AAT CAC AGA GAT
Tfeb	CCA CCC CAG CCA TCA ACA C	CAG ACA GAT ACT CCC GAA CCT T
Ucp1	ACT GCC ACA CCT CCA GTC ATT	CTT TGC CTC ACT CAG GAT TGG

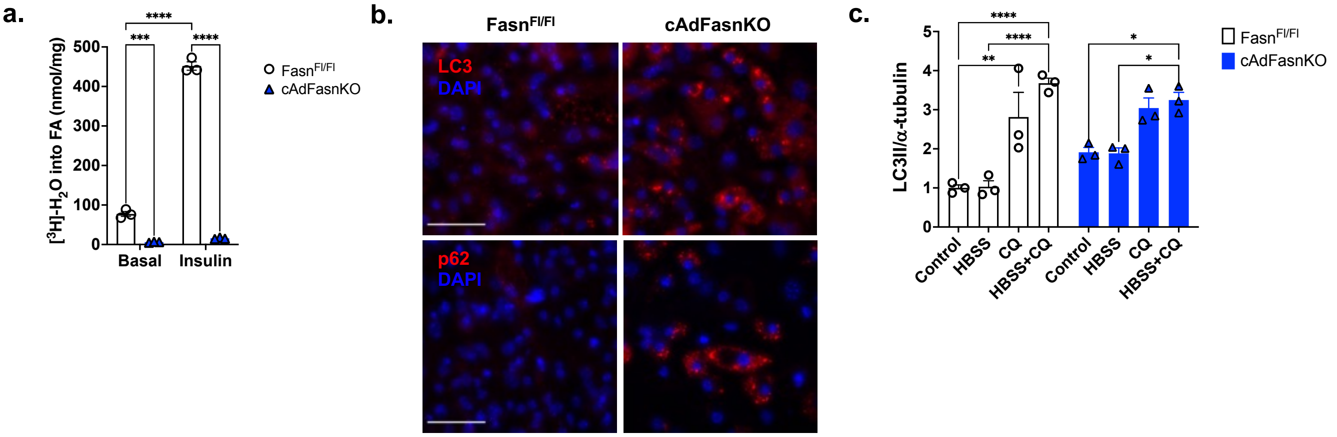

Supplementary Figure 1. Related to Figure 1. Fasn KO adipocytes are deficient in fatty acid synthesis and display impaired autophagy. A) $[^3H]$-H_2O incorporation into fatty acids from in vitro differentiated $Fasn^{Fl/Fl}$ and cAdFasnKO adipocytes under basal conditions and insulin-stimulated conditions. n=3 samples. B) Immunofluorescence of $Fasn^{Fl/Fl}$ and cAdFasnKO adipocytes labeled with LC3 or p62. Scale bar = 50μm. C) Related to Figure 1G. Quantification of LC3II levels normalized to alpha-tubulin. For A and C, 2-way ANOVA with Tukey post hoc, P values = (A) Basal $Fasn^{Fl/Fl}$ vs. basal cAdFasnKO 0.0002, Insulin $Fasn^{Fl/Fl}$ vs. Insulin cAdFasnKO <0.0001, Basal $Fasn^{Fl/Fl}$ vs. Insulin $Fasn^{Fl/Fl}$ <0.0001, Basal cAdFasnKO vs. Insulin cAdFasnKO 0.06852, (C) $Fasn^{Fl/Fl}$: Control vs. CQ 0.0011, Control vs. HBSS+CQ <0.0001, HBSS vs. HBSS+CQ <0.0001, CQ vs. HBSS+CQ 0.1475, cAdFasnKO: Control vs. CQ 0.0406, Control vs. HBSS+CQ 0.0140, HBSS vs. HBSS+CQ 0.0121, CQ vs. HBSS+CQ 0.9489, *<0.05, **<0.01, ***<0.001, ****<0.0001. Source data are provided as a Supplementary Source Data file.

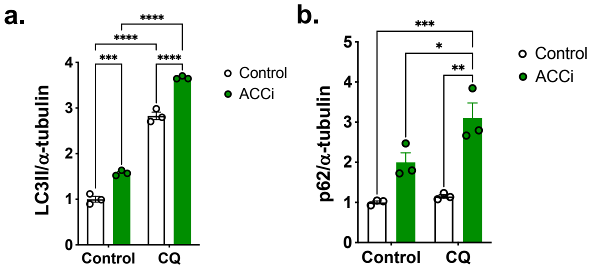

Supplementary Figure 2. Related to Figure 2H. Adipocyte ACC inhibition impairs autophagy. Quantification of soluble LC3II (A) and p62 (B) levels normalized to α-tubulin. n=3 samples, similar data obtained in at least 2 independent experiments. 2-way ANOVA with Tukey post hoc: P values: (LC3II) Main effects: CQ = <0.0001, ACCi = <0.0001, Interaction = 0.0470, Control vs. ACCi = 0.0004, CQ control vs. CQ ACCi = <0.0001, ACCi control vs. CQ ACCi = <0.0001, Control control vs. CQ control = <0.0001, (p62) Main effects: CQ = 0.0228, ACCi = 0.0002, Interaction = 0.0642, Control vs. ACCi = 0.0531, CQ control vs. CQ ACCi = 0.0012, ACCi control vs. CQ ACCi = 0.0328, Control control vs. CQ control = 0.9637, *<0.05, **<0.01, ****<0.0001. All data are means +/- SE. Source data are provided as a Supplementary Source Data file.

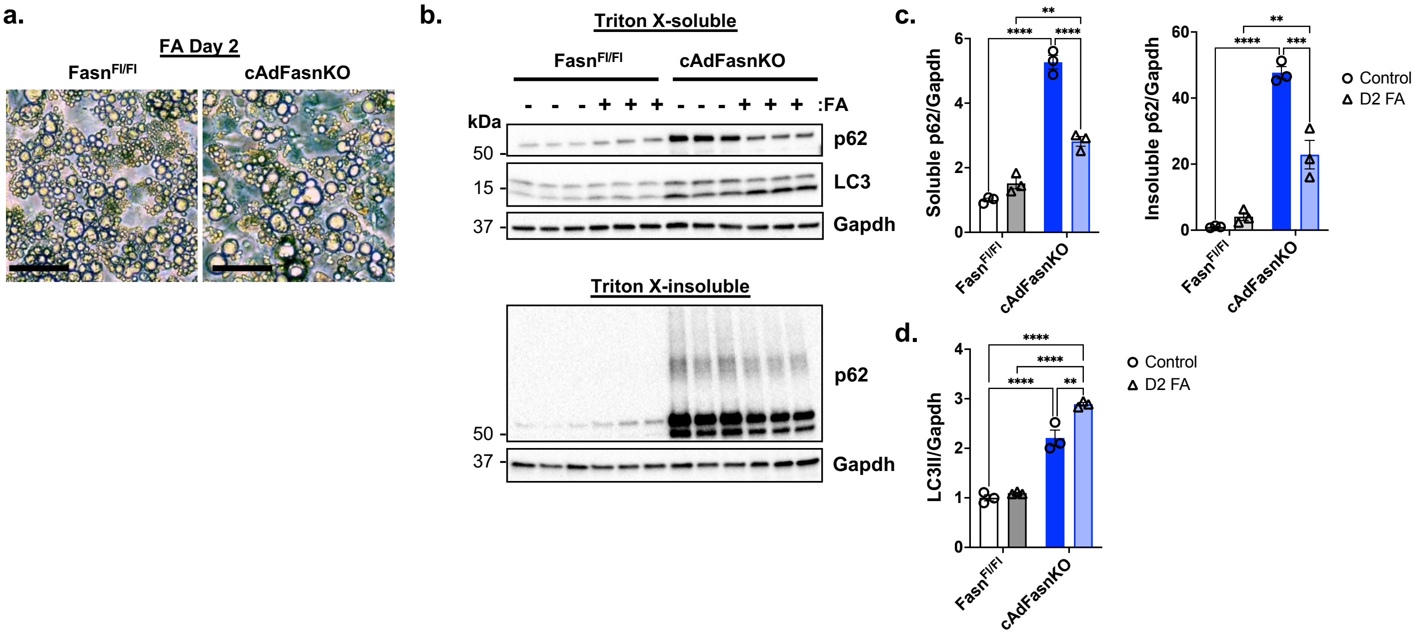

Supplementary Figure 3. Long-term fatty acid supplementation is insufficient to fully restore autophagy in cAdFasnKO adipocytes. A) Light microscopy of Fasn$^{Fl/Fl}$ and cAdFasnKO adipocytes supplemented with 200μM each of palmitate and oleate beginning 48 hours after the onset of differentiation (day 2). Scale bar = 25μm B) Western blots of Triton X-100-soluble and -insoluble protein fractions. C) Quantification of soluble p62 and insoluble p62 from blot in B. D) Quantification of LC3 from blot in B. n=3 samples, similar data obtained in at least 2 independent experiments. Data are means +/- SE. Two-way ANOVA with Tukey post hoc, P values = (C, soluble p62) Fasn$^{Fl/Fl}$ vs. cAdFasnKO <0.0001, Fasn$^{Fl/Fl}$ vs. Fasn$^{Fl/Fl}$ FA 0.1725, Fasn$^{Fl/Fl}$ FA vs. cAdFasnKO FA 0.0017, cAdFasnKO vs. cAdFasnKO FA <0.0001, (C, insoluble p62) Fasn$^{Fl/Fl}$ vs. cAdFasnKO <0.0001, Fasn$^{Fl/Fl}$ vs. Fasn$^{Fl/Fl}$ FA 0.8023, Fasn$^{Fl/Fl}$ FA vs. cAdFasnKO FA 0.0026, cAdFasnKO vs. cAdFasnKO FA 0.0004, (D) Fasn$^{Fl/Fl}$ vs. cAdFasnKO <0.0001, Fasn$^{Fl/Fl}$ vs. Fasn$^{Fl/Fl}$ FA 0.8647, Fasn$^{Fl/Fl}$ FA vs. cAdFasnKO FA <0.0001, cAdFasnKO vs. cAdFasnKO FA 0.0025, **<0.01, ***<0.001, ****<0.0001. Source data are provided as a Supplementary Source Data file.

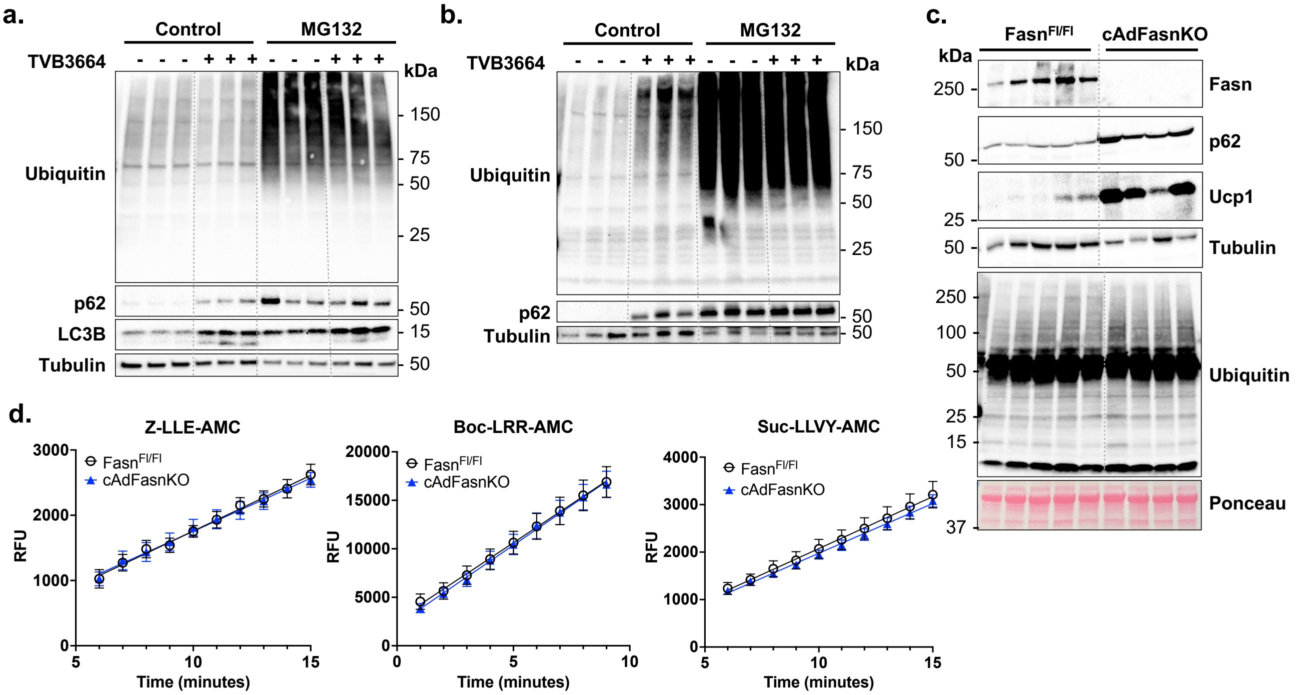

Supplementary Figure 4. General proteostasis is unaltered by Fasn deficiency. Wild-type primary adipocytes were differentiated and treated with 100nM TVB3664 for 48hrs and a subset were treated with 20μM MG132 for 18 hours prior to harvest. A) Western blot of Triton X-100 soluble protein fraction for ubiquitinated proteins, p62, and LC3B. B) Western blot of Triton X-100 insoluble protein fraction for ubiquitinated proteins and p62. n=3 samples (A and B). C) Western blot for Fasn, p62, Ucp1, and Ubiquitinated proteins in $Fasn^{Fl/Fl}$ and cAdFasnKO subcutaneous WAT. D) Proteasomal activity assay performed on lysates from WAT shown in C using the substrates: Z-LLE-AMC (caspase-like), Boc-LRR-AMC (trypsin-like), Suc-LLVY-AMC (chymotrypsin-like). The rates shown are those calculated after subtracting the MG132-inhibited rate and only the linear portions of the curves are shown. n=5 $Fasn^{Fl/Fl}$ mice and n=4 cAdFasnKO mice. Data shown are means +/- SE. Source data are provided as a Supplementary Source Data file.

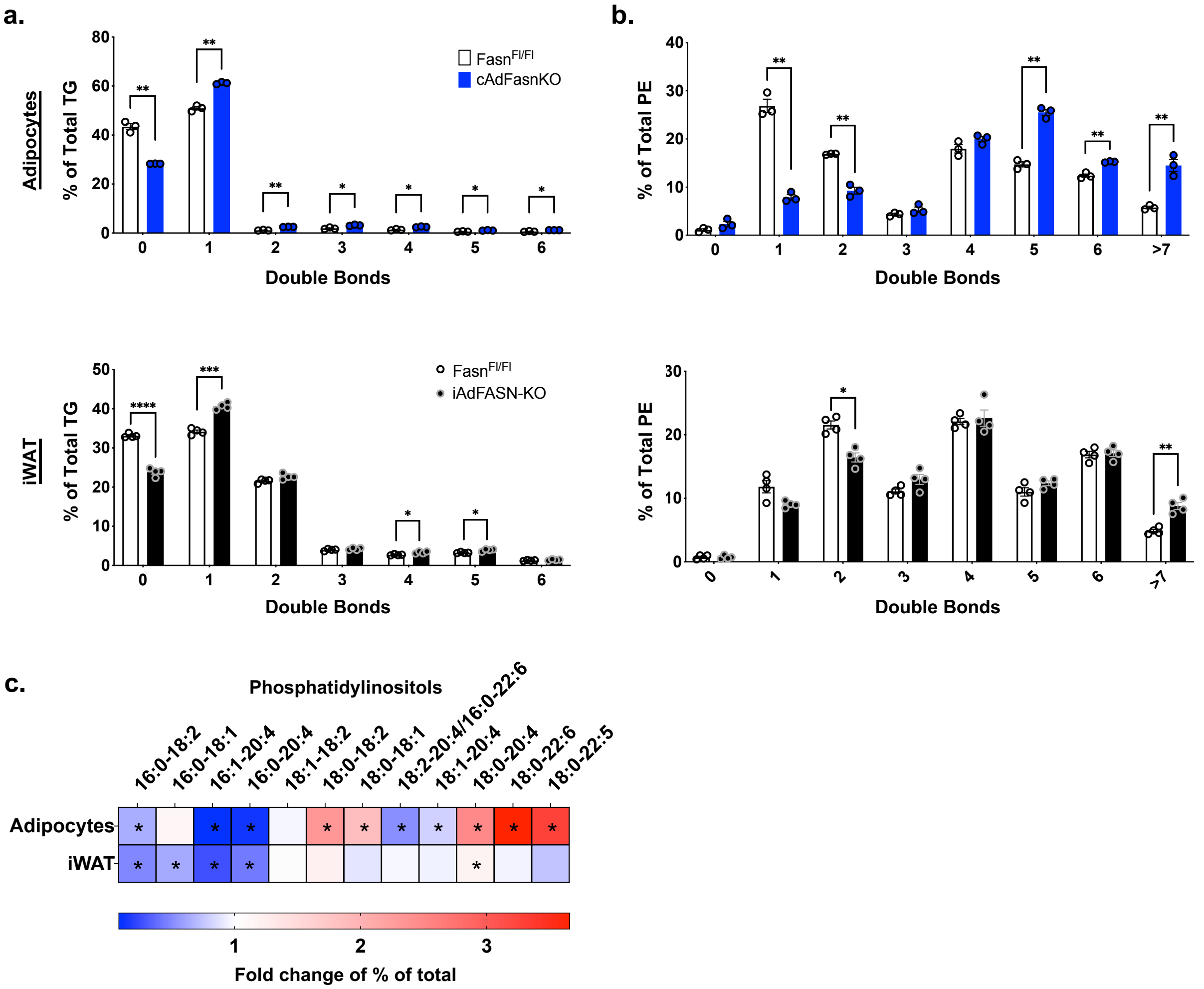

Supplementary Figure 5. Related to Figure 5. Adipocyte Fasn KO alters the fatty acyl composition of lipids in vitro and in vivo. A) Double bond composition of triglycerides and B) phophatidylethanolamine (PE). Top panels are in vitro cultured adipocytes and bottom panels are from in vivo adipose tissue. n=3 samples for in vitro analyses. n=4 mice for in vivo analyses. Data are means +/- SE. Two-tailed t tests: P values = (A, top panel, left to right) 0.001031, 0.001155, 0.015241, 0.015241, 0.015241, 0.015241, (A, bottom panel, left to right) 0.000027, 0.000161, 0.11178, 0.323336, 0.046514, 0.033061, 0.323336, (B, top panel, left to right) 0.325292, 0.001663, 0.002742, 0.325292, 0.325292, 0.001663, 0.006235, 0.009278 (B, bottom panel, left to right) 0.975953, 0.17312, 0.011694, 0.312198, 0.975953, 0.410439, 0.975953, 0.005825, *<0.05, ***<0.001, ****<0.0001. C) Heat map displaying fold changes of the percent of total phosphatidylinositol species from Fasn$^{Fl/Fl}$ vs. cAdFasnKO adipocytes and iAdFasnKO iWAT. Two-tailed t tests, P values = Adipocytes (from left to right) 0.000185, 0.424452, 0.000004, 0.000001, 0.274577, 0.000017, 0.003795, 0.000037, 0.001006, 0.000003, 0.000001, 0.000493, iWAT (from left to right) 0.000472, 0.002971, 0.000409, 0.000722, 0.845883, 0.23546, 0.272081, 0.759332, 0.193383, 0.003495, 0.722369, 0.235208, * <0.05. Source data are provided as a Supplementary Source Data file.

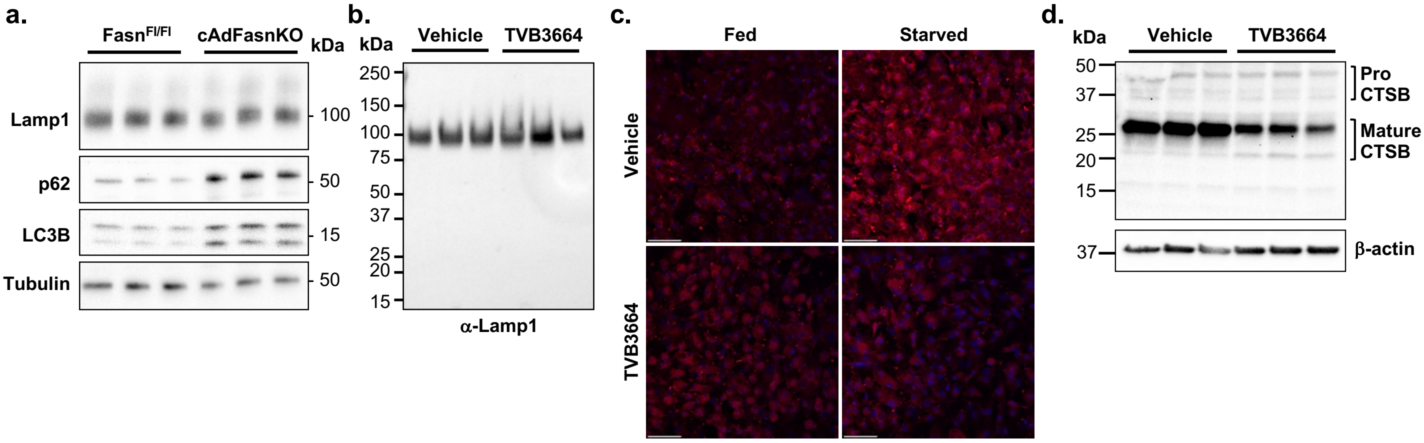

Supplementary Figure 6. Lysosomal activity is impaired by Fasn deficiency. A) Western blot for lysosomal membrane protein Lamp1, p62, and LC3B in Fasn$^{Fl/Fl}$ and cAdFasnKO primary adipocytes. n=3 samples. B) Western blot for Lamp1 in TVB3664-treated primary adipocytes. n=3 samples. C) Magic Red Cathepsin B assay in vehicle or TVB3664-treated adipocytes. Adipocytes were loaded with Magic Red Cathepsin B substrate for 40 minutes in nutrient replete (Fed) media or HBSS (starved). n=3 samples. Scale bar = 50μm D) Western blot for Cathepsin B in TVB3664-treated primary adipocytes. n=3 samples. Source data are provided as a Supplementary Source Data file.

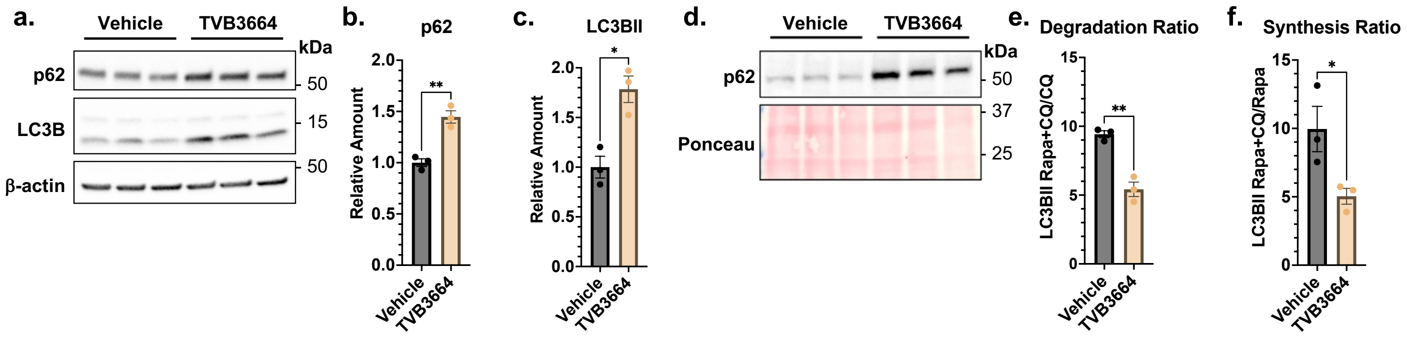

Supplementary Figure 7. Fasn inhibition in HepG2 cells impairs autophagy. HepG2 cells were treated with vehicle or 100nM TVB3664 for 48hrs. A) Western blot of Triton X-100 soluble proteins. B) Quantification of p62 normalized to β-actin from western blot in A. C) Quantification of LC3BII normalized to β-actin from western blot in A. D) Western blot of Triton X-100 insoluble p62. Ponceau S-stained membrane provided as loading control. E) An LC3II turnover assay was performed after 18hr treatment with 5uM rapamycin (Rapa) and 50μM CQ. The degradation ratio was calculated from the change in LC3BII levels in response to the Rapa+CQ treatment compared to CQ alone. F) The synthesis ratio was calculated from the change in LC3BII in response to Rapa+CQ compared to Rapa alone. For B,C, and E, n=3 samples, similar data obtained in at least 2 independent experiments. All data are means +/- SE. Two-tailed t tests, P values = (B) 0.0033, (C) 0.0105, (E) 0.0023, (F) 0.0487, *<0.05, **<0.01. Source data are provided as a Supplementary Source Data file.
