# Supplementary material for: De novo lipogenesis fuels adipocyte autophagosome and lysosome membrane dynamics
Source: Nat Commun. 2023 Mar 13;14:1362. doi: 10.1038/s41467-023-37016-8 (PMC10011520; doi:10.1038/s41467-023-37016-8)
Supplement: Supplementary file 3 — Description of Additional Supplementary Files [file 41467_2023_37016_MOESM3_ESM.pdf]

File name: Supplementary Data 1

Description: Lipidomics datasets from in vitro differentiated Fasn$^{Fl/Fl}$ and cAdFasnKO adipocytes and subcutaneous adipose tissue of Fasn$^{Fl/Fl}$ and iAdFasnKO mice. Lipids were normalized to total protein content.
