# Supplementary material for: De novo lipogenesis fuels adipocyte autophagosome and lysosome membrane dynamics
Source: Nat Commun. 2023 Mar 13;14:1362. doi: 10.1038/s41467-023-37016-8 (PMC10011520; doi:10.1038/s41467-023-37016-8)
Supplement: Supplementary file 5 — Reporting Summary [file 41467_2023_37016_MOESM5_ESM.pdf]

nature portfolio

Corresponding author(s): Michael Czech, Leslie Rowland

Last updated by author(s): Feb 14, 2023

Reporting Summary

Nature Portfolio wishes to improve the reproducibility of the work that we publish. This form provides structure for consistency and transparency in reporting. For further information on Nature Portfolio policies, see our Editorial Policies and the Editorial Policy Checklist.

Statistics

For all statistical analyses, confirm that the following items are present in the figure legend, table legend, main text, or Methods section.

n/a	Confirmed	
☐	☒	The exact sample size (*n*) for each experimental group/condition, given as a discrete number and unit of measurement
☐	☒	A statement on whether measurements were taken from distinct samples or whether the same sample was measured repeatedly
☐	☒	The statistical test(s) used AND whether they are one- or two-sided<br>*Only common tests should be described solely by name; describe more complex techniques in the Methods section.*
☒	☐	A description of all covariates tested
☐	☒	A description of any assumptions or corrections, such as tests of normality and adjustment for multiple comparisons
☐	☒	A full description of the statistical parameters including central tendency (e.g. means) or other basic estimates (e.g. regression coefficient) AND variation (e.g. standard deviation) or associated estimates of uncertainty (e.g. confidence intervals)
☐	☒	For null hypothesis testing, the test statistic (e.g. *F*, *t*, *r*) with confidence intervals, effect sizes, degrees of freedom and *P* value noted<br>*Give P values as exact values whenever suitable.*
☒	☐	For Bayesian analysis, information on the choice of priors and Markov chain Monte Carlo settings
☒	☐	For hierarchical and complex designs, identification of the appropriate level for tests and full reporting of outcomes
☒	☐	Estimates of effect sizes (e.g. Cohen's *d*, Pearson's *r*), indicating how they were calculated

Our web collection on statistics for biologists contains articles on many of the points above.

Software and code

Policy information about availability of computer code

Data collection	Western blotting data: ChemiDoc (Bio-Rad)<br>qPCR data: BioRad CFX97 thermocycler<br>Immunofluorescence data: Leica TCS SP8 confocal microscope<br>Electron Microscopy: Philips CM 10 transmission electron microscope<br>Lipidomics: Triple quadrupole mass spectrometer (TSQ Altis, Thermo Fisher Scientific) and a Q Exactive mass spectrometer (Thermo Scientific), with an automated nanospray device (TriVersa NanoMate, Advion Bioscience Ltd)<br>Metabolomics: Waters Xevo TQ-S triple quadrupole mass spectrometer coupled to a Waters Acquity UPLC system (Waters)
Data analysis	Graphing and statistical analyses: GraphPad Prism (v9.2.0) and Microsoft Excel (v16.66.1)<br>Immunofluorescence analysis: ImageJ (v1.53) and Leica LAS X free version (v3.7.6)<br>Western blotting analysis: Bio-Rad Image Lab (v6.1)<br>Lipidomics: Xcalibur software (Thermo Fisher 4.2.47); identification of lipid species was performed using a home-made software program based on macros programming, patented by Dr. Xianlin Han (doi: 10.1021/ac900241u)

For manuscripts utilizing custom algorithms or software that are central to the research but not yet described in published literature, software must be made available to editors and reviewers. We strongly encourage code deposition in a community repository (e.g. GitHub). See the Nature Portfolio guidelines for submitting code & software for further information.

Data

Policy information about availability of data

All manuscripts must include a data availability statement. This statement should provide the following information, where applicable:

- Accession codes, unique identifiers, or web links for publicly available datasets
- A description of any restrictions on data availability
- For clinical datasets or third party data, please ensure that the statement adheres to our policy

All data generated or analyzed during this study are included with this manuscript and its supplementary information files. Schematic figures were made under a license with BioRender.

Human research participants

Policy information about studies involving human research participants and Sex and Gender in Research.

Reporting on sex and gender	No human subject research was performed in this study.
Population characteristics	N/A
Recruitment	N/A
Ethics oversight	N/A

Note that full information on the approval of the study protocol must also be provided in the manuscript.

Field-specific reporting

Please select the one below that is the best fit for your research. If you are not sure, read the appropriate sections before making your selection.

☒ Life sciences ☐ Behavioural & social sciences ☐ Ecological, evolutionary & environmental sciences

For a reference copy of the document with all sections, see nature.com/documents/nr-reporting-summary-flat.pdf

Life sciences study design

All studies must disclose on these points even when the disclosure is negative.

Sample size	Sample sizes for animal experiments were chosen based on previous experiments performed on each mouse line (doi: 10.1016/j.molmet.2017.05.012). Sample sizes for cell culture experiments were based on previous experience and comparable experiments (doi.org/10.1038/s41467-020-17985-w, doi: 10.1016/j.molmet.2017.05.012)
Data exclusions	Data were only excluded in the event of technical errors.
Replication	All data were replicated a minimum of 2 times with similar outcomes. The lipidomics analyses were only performed once due to the cost of these experiments.
Randomization	The wells of the cell culture plates and mice used in this study were randomly assigned to experimental groups.
Blinding	Data collections and analyses were not blinded due to limitations of available personnel.

Reporting for specific materials, systems and methods

We require information from authors about some types of materials, experimental systems and methods used in many studies. Here, indicate whether each material, system or method listed is relevant to your study. If you are not sure if a list item applies to your research, read the appropriate section before selecting a response.

Materials & experimental systems

n/a	Involved in the study	
☐	☒	Antibodies
☐	☒	Eukaryotic cell lines
☒	☐	Palaeontology and archaeology
☐	☒	Animals and other organisms
☒	☐	Clinical data
☒	☐	Dual use research of concern

Methods

n/a	Involved in the study	
☒	☐	ChIP-seq
☒	☐	Flow cytometry
☒	☐	MRI-based neuroimaging

Antibodies

Antibodies used

For Western blotting and IHC: anti-Fasn (CST #3180); anti-Ucp1 (Abcam #10983); anti-p62 (CST #23214); anti-alpha-tubulin (Sigma #T5168); anti-LC3B (CST #83506), anti-LC3A/B (CST #12741); anti-Gapdh (CST #8884); anti-malonylated lysines (PTM Biolabs #901); anti-vinculin (CST #18799); anti-Gabarap (CST #13733), anti-ubiquitin (Proteintech #10201-2-AP), anti-Lamp1 (BD Biosciences #553792), anti-Cathepsin B (Proteintech #12216-1-AP), anti-b-actin (Sigma #A5316)
For Immunofluorescence: rabbit anti-Fasn (ab22759), rabbit anti-p62 (CST #23214); mouse anti-p62 (R&D #MAB8028); rabbit anti-LC3A/B (CST #12741); mouse anti-LC3B (CST #83506); Alexa Fluor-594 goat anti-rabbit (A-11012) and Alexa Fluor-488 (A-11001) goat anti-mouse (Invitrogen)

Validation

anti-Fasn (CST #3180, C20G5) was validated with the FasnKO adipose tissue and primary adipocytes. The remaining antibodies were validated by the companies from which they were purchased. The datasheets can be found at the following links:
anti-Fasn (CST #3180, C20G5): https://www.cellsignal.com/products/primary-antibodies/fatty-acid-synthase-c20g5-rabbit-mab/3180
anti-Ucp1 (Abcam #10983): https://www.abcam.com/ucp1-antibody-ab10983.html
anti-p62 (CST #23214, D6M5X): https://www.cellsignal.com/products/primary-antibodies/sqstm1-p62-d6m5x-rabbit-mab-rodent-specific/23214
anti-Fasn (Abcam, #ab22759): https://www.abcam.com/fatty-acid-synthase-antibody-ab22759.html
anti-alpha-tubulin (Sigma #T5168, Clone #B-5-1-2): https://www.sigmaaldrich.com/US/en/product/sigma/t5168
anti-LC3B (CST #83506, E5Q2K): https://www.cellsignal.com/products/primary-antibodies/lc3b-e5q2k-mouse-mab/83506
anti-LC3A/B (CST #12741, D3U4C): https://www.cellsignal.com/products/primary-antibodies/lc3a-b-d3u4c-xp-rabbit-mab/12741
anti-Gapdh (CST #8884, D16H11): https://www.cellsignal.com/products/antibody-conjugates/gapdh-d16h11-xp-rabbit-mab-hrp-conjugate/8884
anti-malonylated lysines (PTM Biolabs #901): https://www.ptmbiolabs.com/product/pan-anti-malonyllysine-antibody/
anti-vinculin (CST #18799, E1E9V): https://www.cellsignal.com/products/antibody-conjugates/vinculin-e1e9v-xp-rabbit-mab-hrp-conjugate/18799
anti-Gabarap (CST #13733, E1J4E): https://www.cellsignal.com/products/primary-antibodies/gabarap-e1j4e-rabbit-mab/13733
mouse anti-p62 (R&D #MAB8028, Clone #864807): https://www.rndsystems.com/products/human-mouse-rat-p62-sqstm1-antibody-864807_mab8028
anti-ubiquitin (Proteintech #10201-2-AP): https://www.ptglab.com/products/ubiquitin-Antibody-10201-2-AP.htm
anti-Lamp1 (BD Biosciences #553792, Clone #1D4B): https://www.bdbiosciences.com/en-us/products/reagents/flow-cytometry-reagents/research-reagents/single-color-antibodies-ruo/purified-rat-anti-mouse-cd107a.553792
anti-Cathepsin B (Proteintech #12216-1-AP): https://www.ptglab.com/products/CTSB-Antibody-12216-1-AP.htm
anti-b-actin (Sigma #A5316, Clone #AC-74): https://www.sigmaaldrich.com/US/en/product/sigma/a5316

Eukaryotic cell lines

Policy information about cell lines and Sex and Gender in Research

Cell line source(s)

HepG2 cells were obtained from ATCC.

Authentication

HepG2 cells were not authenticated.

Mycoplasma contamination

Cell lines were not tested for mycoplasma contamination.

Commonly misidentified lines
(See ICLAC register)

There were no commonly misidentified lines used in this study.

Animals and other research organisms

Policy information about studies involving animals; ARRIVE guidelines recommended for reporting animal research, and Sex and Gender in Research

Laboratory animals

C57Bl/6J mice were obtained from Jackson Laboratories. FasnFlox/Flox mice on a C57Bl/6J background were generated by Dr. Clay Semenkovich (Washington University). Adiponectin-Cre (Jax strain #028020) and Adiponectin-Cre-ERT2 (Jax strain #024671) were crossed to FasnFlox/Flox mice to obtain adipose-specific Fasn KO mice and inducible adipose-specific Fasn KO mice, respectively. For

	adult mice experiments, male and female mice were used as denoted in the text and used at 8-12 weeks of age. For primary adipocyte isolations, male and female mice were combined and used at 2-4 weeks of age.
Wild animals	No wild animals used were used in this study.
Reporting on sex	Similar phenotypes for male and female mice were observed so both sexes were used throughout the study based on availability. For primary adipocyte isolations, mixtures of male and female mice were used. For adult animal experiments, the sex of the mice used in the experiment is noted.
Field-collected samples	No field-collected samples were used in this study.
Ethics oversight	All animal experiments were conducted under approval from the UMass Chan Medical School's Institutional Animal Care and Use Committee (IACUC).

Note that full information on the approval of the study protocol must also be provided in the manuscript.
